# Supplementary material for: A novel machine learning based approach for iPS progenitor cell identification
Source: PLoS Comput Biol. 2019 Dec 26;15(12):e1007351. doi: 10.1371/journal.pcbi.1007351 (PMC6932749; doi:10.1371/journal.pcbi.1007351)
Supplement: S1 File — (DOC) [file pcbi.1007351.s001.doc]

**Supplementary materials: A Novel Machine Learning Based Approach for iPS Progenitor Cell Identification**

Haishan Zhang^1, 2¶^, Ximing Shao^3¶^, Yin Peng^4¶^, Yanning Teng^1, #a^, Konda Mani Saravanan^1^, Huiling Zhang^1^, Hongchang Li^3*^, Yanjie Wei^1*^

^1^Joint Engineering Research Center for Health Big Data Intelligent Analysis Technology, Center for High Performance Computing, Shenzhen Institutes of Advanced Technology, Chinese Academy of Sciences, Shenzhen, Guangdong 518055, China

^2^University of Chinese Academy of Sciences, No.19(A) Yuquan Road, Shijingshan District, Beijing 100049, China

^3^Shenzhen Key Laboratory for Molecular Biology of Neural Development, Guangdong Key Laboratory of Nanomedicine, Institute of Biomedicine and Biotechnology, Shenzhen Institutes of Advanced Technology, Chinese Academy of Sciences, Shenzhen, Guangdong 518055, China

^4^Department of Pathology, Shenzhen University School of Medicine, Shenzhen, Guangdong, PR China 518060

^#a^China Merchants Bank Network Technology(Hangzhou) Co., Block B of High-tech Industrial Building, 567 Xincheng Road, Binjiang District, Hangzhou, Zhejiang 310051, China

^*^Corresponding authors: Hongchang Li - [hc.li@siat.ac.cn](mailto:hc.li@siat.ac.cn)(HL); Yanjie Wei - [yj.wei@siat.ac.cn](mailto:yj.wei@siat.ac.cn)(YW)

^¶^These authors contributed equally to this work.

**Part 1: Features and Calculation**

In this paper, features are computed for each identified cell image at different time frames, and these features denote the morphological and movement information of the identified cell during reprogramming. Overall 11 types of features are extracted and the detailed list is as follows:

- *Volume*. Cell volume is calculated by multiplying the number of voxels in the cell image by the volume of each voxel. The volume of voxels is kept constant by Imaris.
- *Area*. Cell surface area is computed as the sum of the surface triangles forming the cell.
- *Sphericity*. Cell sphericity is defined by Wadell and can measure how spherical an object is. It is a ratio of a sphere surface area with same volume as given cell to the given cell surface area. The sphericity Ψ is calculated as follows:

$$\psi=\frac{\pi^{\frac{1}{3}}(6V_{o})^{\frac{2}{3}}}{A_{o}}$$

where *V_o_* represents volume of the object and *A_o_* represents surface area of the object.

- *Ellipsoid-prolate*. Here cells are modeled as ellipsoids where equatorial radii along *x* and *y* axes are set as *b* and *c*, polar radius along *z* axis is set as *a*. Ellipsoid-prolate describes cigar-shaped degree for cells. It is calculated as follows:

$$e_{prolate}=\frac{2a^{2}}{a^{2}+b^{2}}*\left(1-\frac{a^{2}+b^{2}}{2c^{2}}\right)$$

where *a* is a constant 0.5 micron.

- *Ellipsoid-oblate*. Similar to ellipsoid-prolate, ellipsoid-oblate describes disk-shaped degree for cells. It is calculated as follows:

$$e_{oblate}=\frac{2b^{2}}{b^{2}+c^{2}}*\left(1-\frac{2a^{2}}{b^{2}+c^{2}}\right)$$

where *a*, *b* and *c* are same as above.

- *Nucleus - cytoplasm volume ratio*. Nucleus - cytoplasm volume ratio is an important indicator for cell cycle and development. It can be an effective feature for classification since iPS progenitor cells show decrease in size during early reprogramming[1]. The value is defined as follows:

$$ratio=\frac{the\text{ }volume\text{ }of\text{ }nuclei}{(the\text{ }whole\text{ }cell\text{ }volume-the\text{ }volume\text{ }of\text{ }nuclei)}$$

- *Speed*. Speed of a cell is its instant speed at the time frame when the cell image is taken. The speed is defined as follows:

$$Speed(t)=\frac{\sqrt{D_{x}(t,t-1)^{2}+D_{y}(t,t-1)^{2}+D_{z}(t,t-1)^{2}}}{T(t)-T(t-1)}$$

where *T(t)* represents time in seconds at frame *t*. *D(t, t-1)* denotes the displacement of the cell from time *t-1* to time *t* in *x*, *y* or *z* direction. It is defined as follows:

$$D_{i}(t,t-1)=P_{i}(t)-P_{i}(t-1),i=\{x,y,z\}$$

where *P_i_(t)* represents i-position of object at frame *t*.

- *Displacement*. The value represents the displacement of the cell centroid from its current time frame to the starting/initial frame.
- *Intensity-stdDev*. The value represents the voxel intensity standard deviation calculated from all voxel intensity values in the identified cell.
- *Intensity-Max*. The value represents the maximum voxel intensity among all voxels identified in the cell.
- *Intensity-Min*. The value represents the minimum voxel intensity of all voxel intensity contained in the cell.

**Part 2: Supplementary Figure**

Fig A: the precisions for six missing frame numbers and three imputation methods based on six time periods (TP1~TP6).


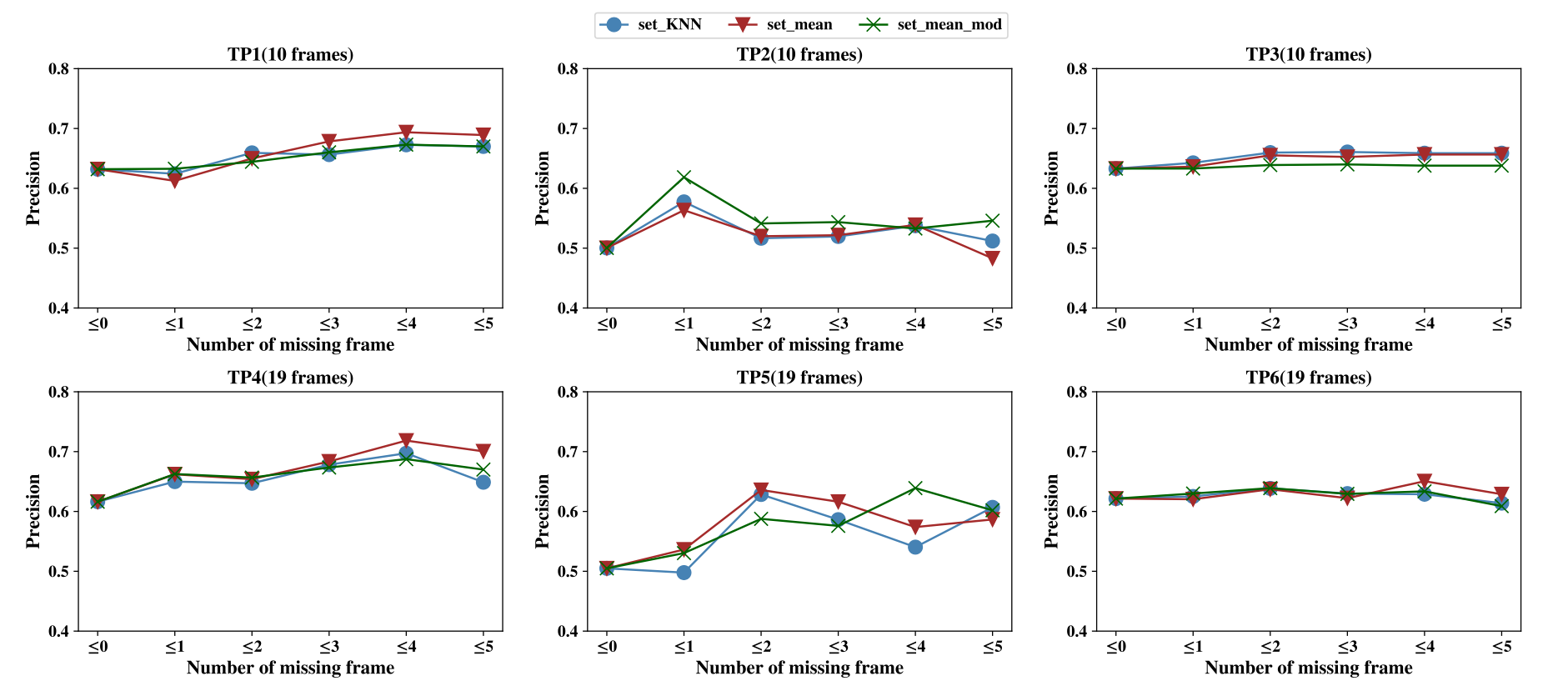


**Part 3: Supplementary Tables**

Table A: Precision values, average precisions and the corresponding standard deviation (STD) of 3 different best start frames.

| Frame No. | Prec(Phase 1) | Prec (Phase 2) | Prec. (Phase 3) | Ave Prec. | STD |
| --- | --- | --- | --- | --- | --- |
| 11 | 0.65 | 0.57 | 0.68 | 0.63 | 0.06 |
| 13 | 0.66 | 0.64 | 0.65 | 0.64 | 0.01 |
| 15 | 0.61 | 0.62 | 0.60 | 0.60 | 0.01 |
| 17 | 0.56 | 0.63 | 0.65 | 0.61 | 0.04 |

**Reference**

1. Smith ZD, Nachman I, Regev A, Meissner A. Dynamic single-cell imaging of direct reprogramming reveals an early specifying event. Nat Biotechnol. 2010;28(5):521-6.
